# Graphene-Based Raman Spectroscopy for pH Sensing of X-rays Exposed and Unexposed Culture Media and Cells

**DOI:** 10.3390/s18072242

**Published:** 2018-07-12

**Authors:** Carlo Camerlingo, Alessandro Verde, Lorenzo Manti, Roberta Meschini, Ines Delfino, Maria Lepore

**Affiliations:** 1 Consiglio Nazionale delle Ricerche, SPIN-CNR, 80078 Pozzuoli, Italy; 2 Dipartimento di Fisica “E. Pancini”, Università “Federico II” di Napoli, 80126 Naples, Italy; alessa.verde@studenti.unina.it (A.V.); lorenzo.manti@unina.it (L.M.); 3 Dipartimento di Scienze Ecologiche e Biologiche, Università della Tuscia, 01100 Viterbo, Italy; meschini@unitus.it (R.M.); delfino@unitus.it (I.D.); 4 Dipartimento di Medicina Sperimentale, Università della Campania “L. Vanvitelli”, 80138 Naples, Italy; maria.lepore@unicampania.it

**Keywords:** graphene, micro-Raman spectroscopy, culture media, human cells, physiometer bio-sensors

## Abstract

Graphene provides a unique way of sensing the local pH level of substances on the micrometric scale, with important implications for the monitoring of cellular metabolic activities where proton excretion could occur. Accordingly, an innovative biosensing approach for the quantification of the pH value of biological fluids, to be used also with small amounts of fluids, was realized and tested. It is based on the use of micro-Raman spectroscopy to detect the modifications of the graphene doping level induced by the contact of the graphene with the selected fluids. The approach was preliminarily tested on aqueous solutions of known pH values. It was then used to quantify the pH values of cell culture media directly exposed to different doses of X-ray radiation and to media exposed to X-ray-irradiated cells. The Raman response of cells placed on graphene layers was also examined.

## 1. Introduction

Graphene is a new generation material, which is the basic structural element of other allotropes of carbon such as graphite, charcoal and fullerenes. Its peculiar properties (heat and electric conductivity, optical properties, elasticity) make it a useful material for scientific applications in a wide variety of scientific fields [1,2,3,4,5,6,7,8]. The electronic properties of graphene are due to its atomic structure. In fact, its carbon atoms present a particular organization that permits the formation of π and $\pi^{*}$ bands so that electrons are free to move along the material surface [9]. On the other hand, thanks to these half-filled bands, charge carriers deposited on graphene’s surface are able to change the atomic structure of the material by the addition or the subtraction of valence electrons [10]. In the biomedical field, graphene electronic properties can be exploited to investigate cells and biological fluids in order to develop a sensing scheme useful for diagnostic applications [11,12,13,14]. In addition, in recent years, Raman spectroscopies (resonance Raman, surface-enhanced Raman) have been fruitfully employed to study biological materials, including biofluids, cells and tissues [15,16,17,18,19,20]. The potentiality of graphene to sense the acidity of substances on the micrometric scale was proven by G. L. C.Paulus and coworkers [21]. They proposed to use Raman spectroscopy to measure graphene *p*-doping [22] in order to monitor local pH changes, opening amazing perspectives in the field of bio-cellular investigation. In the present work, the proposed approach has been applied to the study of cell culture media in different conditions. This class of biological samples has been largely investigated in the last few years using Raman and surface enhanced Raman (SERS) spectroscopies in order to identify different culture media [23], to study recombinant antibody production [24], to monitor their degradation processes [25] and metabolites’ dynamics [26]. Two culture media irradiated with different doses of X-rays were herein examined in order to investigate the induced differences. In addition, the changes in graphene *p*-doping due to the interaction with human cells placed on the graphene layer were also considered.

## 2. Materials and Methods

Single-layer graphene on 10×10 mm${}^{2}$ Si substrates was used in this work. These were produced by Graphenea (San Sebastiàn, Spain). In order to calibrate the sensor, aqueous solutions were prepared with nominal pH equal to 2, 4.5, 7.4, 10, 10.7 and 12 and tested on graphene. Using ultrapure deionized water (pH = 7.4), HCl (3 molars) or NaOH (2 molars) solutions were diluted in order to obtain unbuffered solutions with a known concentration and pH. Two different Dulbecco’s Modified Eagle Media (DMEM-A and DMEM-B) were investigated. DMEM-A (Microtech${}^{\mathrm{TM}}$ research (Italy)) is a high glucose cell culture medium supplemented with 10% FBS (Fetal Bovine Serum), 1% L-glutamine and 1% streptomycin/penicillin. Samples of DMEM-A were tested after different exposure times to X-ray ionizing irradiation processes performed at a dose rate of 2.1 Gy/min. Five different doses equal to 2, 4, 6, 8 and 10 Gy, respectively, were considered together with unexposed medium (0 Gy). The X-ray irradiation treatment was performed using a STABILIPLAN machine (Siemens, Munich, Germany). X-rays (250 kVp) were produced by a Thomson tube (TR 300 F) and filtered by 1 mm thick foil. DMEM-B samples (GibcoTM Dulbecco Modified Eagle Medium, by Thermo Fisher Sci. Inc., Waltham, MA, USA; cell culture media supplemented with 15% FBS, 1% L-glutamine, 1% streptomycin/penicillin and 0.1% gentamicin) were extracted from cultures of the SH-SY5Y cell line irradiated at different doses of 2 and 4 Gy with a Gilardoni MGL 200/8 D machine (Milan, Italy) that operates at 250 kV and 6 mA (dose rate 60 cGy/min). After the irradiation, the cells were stored in an incubator for 24 h, and then, the medium was extracted from the cell cultures; DMEM samples with no contact with cells and samples of DMEM put in contact with unexposed cells were also available. The SH-SY5Y neuroblastoma cell culture line (American Type Culture Collection, Manassas, VA, USA) is representative of the most common cancer in infants and the third most common cancer in children, after leukemia and brain cancer.

The cells examined on graphene substrates belong to the MDA-MB-231 cell line (they were a gift by L. Minafra, IBFM-CNR, Cefalù, Italy). This cell line is a highly aggressive, invasive and poorly-differentiated Triple-Negative Breast Cancer (TNBC) cell line. After sterilizing a graphene film by briefly (about 10 min) submerging it in ethanol, the sample was rinsed thoroughly with a sterile saline solution (PBS) to remove any residual ethanol, which is cytotoxic, and placed in a 30 mm Petri dish. Zero-point-five milliliters of the cell culture suspension were further diluted with 4.5 mL of medium, and then, this was deposited in the Petri dish, where the graphene substrate lied. The cells were then placed in an incubator for 24 h in order to let them adhere to the graphene film.

**Figure 1:**
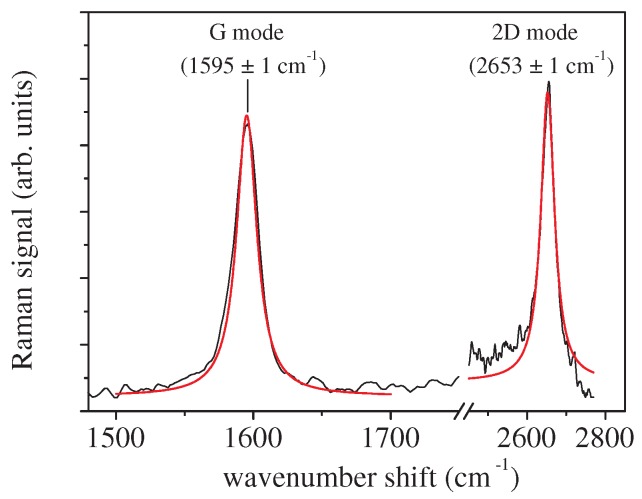
Raman spectrum of pristine graphene. The two main Raman modes (G and 2D modes) have been fitted by Lorentzian functions (red lines) centered at 1595 ± 1 cm${}^{-1}$ and at 2653 ± 1 cm${}^{-1}$, respectively.

For all the samples, a drop of about 1 μL was considered for the Raman analysis. Micro-Raman spectroscopy was performed by using a Jobin-Yvon system (by Horiba Inst. Ltd., Kyoto, Japan) equipped with a TriAx 180 monochromator, a liquid N${}_{2}$-cooled CCD and an optical grating of 1800 grooves/mm, allowing a spectral resolution of 4 cm${}^{-1}$. A He-Ne laser operating at a wavelength λ = 633 nm was used (maximum nominal power of 17 mW). The laser light was focused on the sample surface by means of a 100× (n.a. = 0.90) optical objective on an excitation area of about 1 μm in size. The spectra were obtained using an accumulation time of 180 s. All the measurements were performed at least three times, in different positions of the samples, and the results were averaged. The spectra were numerically pre-processed in order to remove the background signal, and the peak characteristics were determined by a fitting procedure, modeling the peaks with Lorentzian functions (see Figure 1).

## 3. Results and Discussion

### 3.1. Aqueous Solutions at Different pH

The Raman response of graphene in contact with substances with different pH values was investigated by considering aqueous solutions of HCl or NaOH in the pH range from 2–14. The Raman signal of bare graphene was characterized by a sharp feature at about 1595 cm${}^{-1}$ (G mode) and a broad mode at 2653 cm${}^{-1}$ (2D mode) (Figure 1) [27].

**Figure 2:**
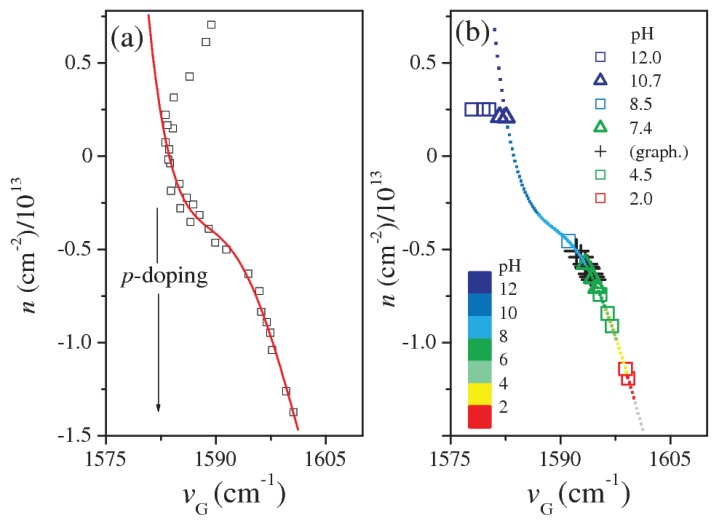
(**a**) An empirical analytical relation between *n* and $\nu_{G}$ has been obtained by fitting the experimental data (squares) taken from [17]. (**b**) Estimations of the doping carrier density *n* for aqueous solutions at different nominal pH levels (pH = 2.0, 4.5, 7.4, 8.5, 10.7, 12.0). Black crosses refer to pristine graphene (with air contact). A pH scale is empirically assigned to the *n* dependence on $\nu_{G}$ and graphically shown by the color mapping.

The measured position of the G mode ($\nu_{G}$) was higher than the value expected in floating graphene. This was caused by an initial *p*-doping of graphene due to the contact with the Si substrate and air [21]. The spectral position of the G peak was strongly correlated with the doping degree of the graphene. The excess or defect of charge implied a change of the equilibrium lattice parameters and phonon dispersion, resulting in a shift of the Raman modes [22]. A stiffening of the G mode occurred both in the case of hole (*p*-doping) and electron (*n*-doping) increase. Thus, an estimation of the electron carrier area density *n* could be obtained from the spectral position $\nu_{G}$ of the G mode. The doping changes induced by the contact with acid/alkaline substance were typically confined in the regime of *p*-doping. In this regime, the carrier density *n* was negative (hole-carriers), and its dependence on $\nu_{G}$ was approximated in the spectral range of 1582–1602 cm${}^{-1}$ by an analytical relation obtained by fitting with a 5th order polynomial (see Figure 2a) the experimental data taken from the work of A. Das et al. [22], where the electrochemically-induced *n*- (*p*-) doping changes in graphene were investigated.

**Figure 3:**
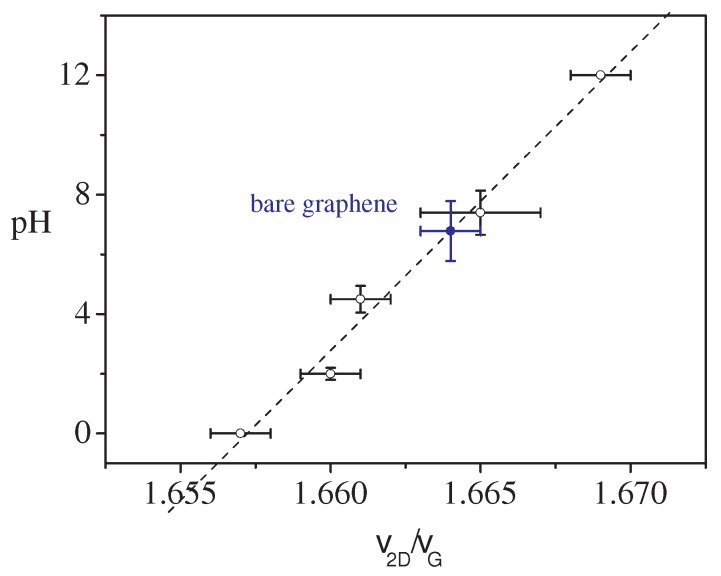
Dependence of the pH value on the ratio between the spectral position of 2D and the G Raman modes detected from the Raman spectra of aqueous solutions with different pH levels (0, 2.0, 4.5, 7.4, 12.0). The data referring to pristine graphene are also reported (blue symbol).

We used this relation for evaluating *n* from the wavenumber position $\nu_{G}$ of the G mode. In Figure 2b, the evaluated *n* is reported as a function of the $\nu_{G}$ position, for the solutions at different nominal pH levels (pH = 2.0, 4.5, 7.4, 8.5, 10.7, 12.0). We obtained an empirical relation between *n* and pH, on the basis of the pH nominal value of the solutions, which has been represented in Figure 2b by the data color map:

(1) $$pH\approx c_{0}+c_{1}n+c_{2}n^{2}$$

with $c_{0}$ = 11.5±0.5, $c_{1}$ = $\left(3.6\pm 1.4\right)\times 10^{-13}$ cm${}^{-2}$ and $c_{2}$ = $\left(-3.9\pm 1.4\right)\times (10^{-13}$ cm${}^{-2}$)${}^{2}$, respectively. The $\nu_{G}$ values decreased (spectral red-shift) when *p*-doping decreases, i.e., when the pH increased. For high alkaline levels (pH > 12), the graphene doping level was low and the carrier density *n* changes strongly depended on the $\nu_{G}$ position (see Figure 2a). The accuracy of pH determination by Equation (1) was smaller than in the other cases (lower pH values). Furthermore, very low values of $\nu_{G}$ have been found for the pH = 12 aqueous solution (Figure 2b), which may have been due to spurious effects related to the degradation of the graphene. Furthermore, the 2D mode was affected by the pH of solutions, the changes in the spectral band position ($\nu_{2D}$) being strongly correlated with $\nu_{G}$. In Figure 3, the ratio $\nu_{2D}$/$\nu_{G}$ is reported for different nominal pH levels. A linear correlation between pH and the $\nu_{2D}$/$\nu_{G}$ ratio was evinced:

(2) $$pH\approx \left(1002\pm 72\right)\times \left(\frac{\nu_{2D}}{\nu_{G}}-1.657\right)$$

The $\nu_{2D}$/$\nu_{G}$ value for bare graphene is reported in Figure 3 (blue symbol) for comparison. It was obtained by averaging the data from Raman spectra acquired in different positions of the graphene substrate area. The resulting $\nu_{2D}$/$\nu_{G}$ ratio value was 1.664 ± 0.001, corresponding to pH = 6.8 ± 1.0, consistent with the neutral condition (pH = 7.0). The average value of $\nu_{G}$ was equal to 1593.7 ± 0.3 cm${}^{-1}$, corresponding to a doping carrier density $n_{g}$ = $-0.60\times 10^{13}$ cm${}^{-2}$. From this $n_{g}$ value, an equivalent pH = 7.9 ± 1.0 was estimated by using Equation (1). In order to overcome errors arising from the variations of the initial doping of the graphene substrate, due to air humidity exposition or aging effects, the Raman measurements on samples were always compared with the data of pristine graphene, acquired on the same substrate, at a point close to the investigated area. The actual doping carrier density $n^{*}$, to be used in Equation (1), results:

(3) $$n^{*}=n-\Delta n_{g}$$

where *n* is the carrier density evaluated for the sample and for the bare graphene reference and $\Delta n_{g}$ is the doping variation of graphene with respect to the value $n_{g}$ found during the calibration procedure.

### 3.2. Irradiated Cell Culture Media

#### 3.2.1. DMEM-A Results

**Figure 4:**
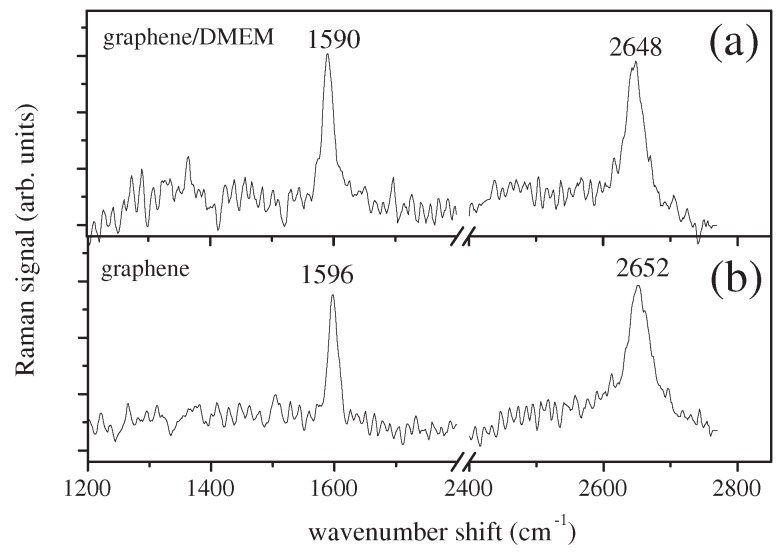
(**a**) Raman spectrum of graphene covered by the unexposed DMEM-A sample (0 Gy). (**b**) Raman spectrum of bare graphene.

Samples of DMEM-A exposed to ionizing radiation, for doses of 2, 4, 6, 8 and 10 Gy, were considered along with unexposed sample (dose of 0 Gy). The Raman response of graphene covered by a drop of unexposed DMEM is reported in Figure 4a. When this spectrum was compared with the Raman spectrum of pristine graphene, a spectral red-shift of the Raman mode at 1590 cm${}^{-1}$ (G mode) and at 2648 cm${}^{-1}$ (2D mode) was clearly observed. The evaluated values for pH were 10.5 ± 1.3, by using Equation (1), and 8.2 ± 1.0, by Equation (2), respectively. These values were higher than the value of 7.4 measured by a conventional pH-meter on the pristine DMEM-A. This result was probably related to a chemical interaction between graphene, DMEM-A and air contact. The DMEM-A was buffered with a high CO${}_{2}$ level. The presence of sodium bicarbonate yielded a basic level increase when the CO${}_{2}$ went away. A further investigation of this aspect is in progress.

**Figure 5:**
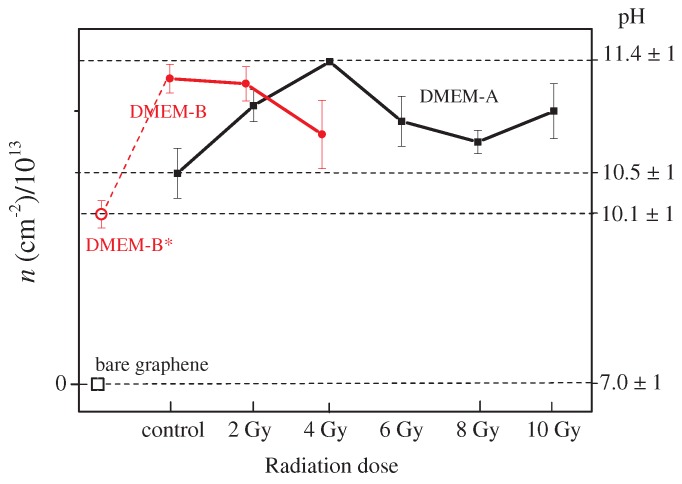
Left axis: Change of carrier doping of graphene in contact with DMEM-A irradiated by X-rays at doses of 0, 2, 4, 6 and 8 Gy (black symbols) and with DMEM-B irradiated at 0, 2 and 4 Gy (red symbols), evaluated by micro-Raman spectroscopy. The data are compared with the intrinsic doping level of bare graphene (pH = 7.0) and DMEM-B${}^{*}$ (samples without contact with cells). Right axis: Evaluated pH values of the investigated culture media.

The value of *n* was estimated also in the case of irradiated DMEM-A samples. The data reported in Figure 5 indicate the occurrence of changes in the pH level with dose. The level of pH increased with irradiation dose and reached a pH value equal to 11.4 ± 1.0 at a dose of 4 Gy. At doses higher than 4 Gy, the pH of DMEM-A decreased gradually, until 8 Gy, then increased again at the dose of 10 Gy. This behavior could be ascribed to an increase of hydroxyl ions (OH${}^{-}$), which at a low dose overcome the H${}^{+}$, resulting in a lower level of the total acidity. Increasing the radiation dose, the content of H${}^{+}$ and OH${}^{-}$ became closer and the pH value decreased. In Table 1, other details about the results of the analysis of the spectra from the DMEM-A samples are reported. The Δn carrier doping changes with respect to the pristine graphene value are listed, together with the $\nu_{2D}$/$\nu_{G}$ ratio and the pH values obtained using Equations (1) and (2). The data showed a trend similar to the ones observed in Figure 5. In addition, the pH values obtained using Equations (1) and (2) were consistent within the estimated errors in almost all cases. It is worth noting that the two considered empirical relations refereed to two different scattering mechanisms. The first one was related directly to phonon vibrational properties of carbon atoms, while in the second case, electron excitation levels were also involved in a two-phonon excitation mechanism [27]. Some discrepancies between the two methods when additive and uncontrolled effects (such as chemical ones) occurred could have originated from this fact. Nevertheless, the agreement between the two methods remained relatively good, and the pH determinations were consistent within the error range.

#### 3.2.2. DMEM-B Results

In Figure 5, the pH values as obtained by analysis of the Raman spectra of DMEM-B samples are also reported. These samples were extracted from cultures of the SH-SYSY cell line irradiated at different doses of 2 and 4 Gy, as previously explained in the Materials and Methods Section. In the graph, also the result for samples not in contact with cells is reported (DMEM-B${}^{*}$). The details of the spectral analysis are reported in Table 2. A larger difference was found between the pH values obtained from Equations (1) and (2), respectively, with respect to estimations obtained for the DMEM-A case. The pH values of the two different DMEM media were consistent within the estimated error; conversely, DMEM-B samples showed a behavior different from DMEM-A samples. This could be ascribed to cell interaction that modified the proton exchanges in the culture medium due to the so-called bystander effect [28].

The radiation-induced bystander effect is the phenomenon for which radiobiological effects are also present in un-irradiated cells in the vicinity of cells that have been irradiated. This is in contrast to the central dogma of radiation biology, affirming that ionizing radiation must interact with the DNA of the cell in order to produce radiobiological effects. Experimental evidence of this effect has been widely observed using different ionizing radiation [29,30]. The precise mechanisms governing how this effect occurs remain unknown, but it is thought to occur via secretion of factors from the irradiated cell that interact with the un-irradiated cell.

### 3.3. Human Cells

**Figure 6:**
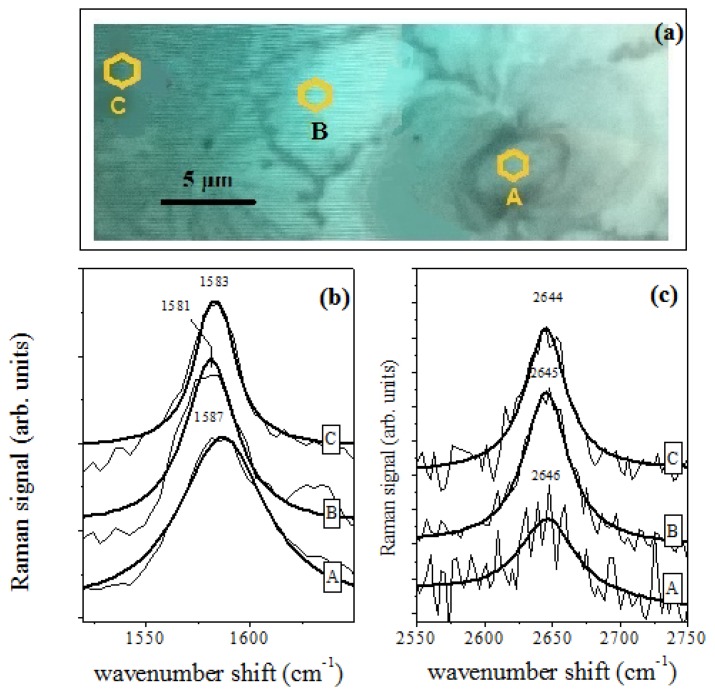
(**a**) Pictures of the Raman acquisition positions (yellow areas) at different distances from the cell center ( Position A). (**b**) G mode Raman spectra acquired in the A, B and C positions. The data are arbitrarily shifted along the y-axis. (**c**) 2D mode Raman spectra acquired in the A, B and C positions. The pH value estimates are 7.8, 10.0 and 9.1, in the A, B and C positions, respectively.

Measurements were performed on MDA-MB-231 cells placed on graphene film, both on the cells and the nearby areas, as schematically shown in the microscope image of Figure 6a. A single cell, placed on the graphene substrate, was selected (Position A in Figure 6a) and investigated.

**Figure 7:**
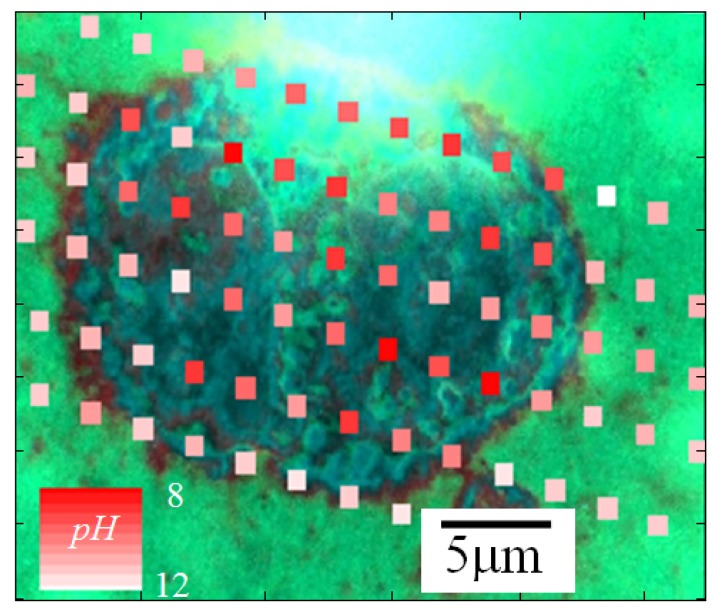
pH evaluation of a single fixed cell by the Raman response of the graphene. The cell area has been scanned at the micrometric scale. Color scale range between pH = 8 (red) and 12 (white).

A single cell, placed on the graphene substrate, was selected (Position A in Figure 6a) and investigated. As can be seen from Figure 6b, the values of the G and of the 2D peak positions changed depending on the distance from the cell. In the spectrum detected from the cell (A position), the G peak was located around 1587 cm${}^{-1}$. When the laser spot was moved away from the cell, the G peak downshifted to 1581 cm${}^{-1}$ (B position) and 1583 cm${}^{-1}$ (C position). Similarly, the 2D peak position slightly decreased as the distance from the cell center increased, shifting from 2646 cm${}^{-1}$ (A position) to 2645 cm${}^{-1}$ (B position) and 2644 cm${}^{-1}$ (C position). As for the DMEMs, the G shift values of the cell could be related to its *n* concentration by using the relation of Figure 2b, and the pH values inside the cell area could be estimated. Using Equation (1), the pH value for the cell was estimated to be equal to 7.8 ± 1.0 (A position). Out of the cell area, the pH changed to 10.0 ± 1.0 (Position B) and 9.1 ± 1.0 (C position). The lower level of alkalinity inside the cell area was compatible with the expected proton emission [28,29,30]. It is important to note that the graphene-based Raman spectroscopy herein adopted can be also applied for pH sensing at the subcellular level, as shown in Figure 7. In this case, the pH level of a single MDA-MB-231 cell placed on graphene substrate was measured in different locations distant from each other by ≈ 2 μm. The pH level ranged between eight and 12.

## 4. Conclusions

Based on the unique properties of graphene, the dependence of the electronic doping of this material induced by the proximity of alkaline or acid liquids was probed by micro-Raman spectroscopy and exploited for a new pH-sensing approach. The method allows the determination of the pH level of solutions even when very small volumes are considered (smaller than 1 μL) or when a high spatial resolution (of order of 1 μm) is required, thanks to the possibility to perform a scan of the sample. pH changes in culture media (DMEM-A and DMEM-B) exposed to X-rays or to irradiated cells were evaluated depending on the X-Ray irradiation doses. The present sensing method could be an additional approach for investigating the radiation-induced bystander effect. In fact, the determination of pH level changes could be extremely useful for elucidating the complex behavior of water hydrolysis induced by radiation [31], which mainly determines the pH level of the sample by regulating the amount of hydroxyl and hydrogen ions.

## Figures and Tables

**Table 1:** Experimental results for DMEM-A exposed to different doses of X-ray radiation.

Irradiation Dose (Gy)	$\nu_{G}$ (cm${}^{-1}$)	$\nu_{2D}$ (cm${}^{-1}$)	$\nu_{2D}$/$\nu_{G}$	$\Delta n/10^{13}$ (cm${}^{-2}$)	pH (Equation (1))	pH (Equation (2))
0	1588.2	2649.1	1.668	0.39±0.20	10.5	8.2
2	1586.6	2647.4	1.669	0.51±0.17	11.1	11.7
4	1587.2	2648.1	1.670	0.59±0.18	11.4	12.7
6	1588.2	2647.1	1.667	0.48±0.20	11.0	9.7
8	1586.8	2647.4	1.667	0.44±0.18	10.8	9.7
10	1586.1	2646.1	1.668	0.50±0.16	11.0	10.7

The error in the pH evaluation is of the order of ±1.0.

**Table 2:** Experimental results for DMEM-B extracted cultures of the SH-SYSY cell line exposed to different X-ray doses. DMEM-B${}^{*}$ indicates samples that had no contact with cells.

Irradiation Dose (Gy)	$\nu_{G}$ (cm${}^{-1}$)	$\nu_{2D}$ (cm${}^{-1}$)	$\nu_{2D}$/$\nu_{G}$	$\Delta n/10^{13}$ (cm${}^{-2}$)	pH (Equation (1))	pH (Equation (2))
DMEM-B*	1584.1	2646.7	1.671	0.31±0.16	10.1	13.8
0	1585.8	2649.6	1.668	0.56±0.21	11.3	13.9
2	1584.3	2646.8	1.671	0.55±0.25	11.8	13.7
4	1587.8	2649.6	1.669	0.49±0.25	10.9	11.7

The error in the pH evaluation is of the order of ±1.0.
